# Long-Term Effects and Predictors of Outcome of Child-Parent Psychotherapy for Traumatized Young Children and their Caregivers: A 6-Month Follow-Up of a Swedish Clinical Sample

**DOI:** 10.1007/s40653-025-00779-x

**Published:** 2025-10-27

**Authors:** Anna Norlén, Charlotte Bäccman, Karin Lindqvist, Jakob Mechler, Agneta Thorén, Kjerstin Almqvist

**Affiliations:** 1 Department of Social and Psychological Studies, Karlstad University, Universitetsgatan 2, Karlstad, 651 88 Sweden; 2 The Erica Foundation, Odengatan 9, Stockholm, 114 24 Sweden; 3 Stockholm University, Frescativägen 8, Stockholm, 106 91 Sweden; 4 Uppsala University, Von Kraemers allé 1 A & 1C, Uppsala, 751 42 Sweden

**Keywords:** Childhood trauma, Child-Parent psychotherapy, Attachment-based therapy, Post-traumatic stress, Long-term effects, Predictors

## Abstract

Children under the age of six are disproportionately exposed to traumatic experiences and seem especially vulnerable. Trauma often affects both children and caregivers and their relationships. Trauma-focused treatment and its long-term effects for young children are of prime interest, but research is limited and lacks follow-up data. The current study explored the long-term effects of Child-Parent Psychotherapy (CPP) treatment and potential predictors of outcome. The sample included 37 traumatized young children, aged 2–6 years, who had received the dyadic treatment together with their caregiver in a multi-site clinical setting. The majority had been exposed to several potential traumatic events, including interpersonal trauma. The study was a naturalistic one-group, pre-post design study with a 6-month follow-up. Outcome measures comprised child and caregiver post-traumatic stress symptoms and signs of caregiving disorganization reported by caregivers. Piecewise Linear Mixed Models were applied to explore long-term treatment effects. Within-group effect sizes were calculated using model-estimated differences in mean values. Possible predictors of outcome were analyzed by adding them as covariates in the model and interacting them with time. The outcomes remained consistent six months after treatment. Positive effects were reduced child and caregiver post-traumatic stress symptoms (*d* = 0.62; *d* = 0.57, respectively) and signs of caregiving disorganization (*d =* 0.64). A higher degree of child trauma symptoms predicted less reduction in caregiver traumatic stress. The results indicate that children, caregivers, and their relationship benefit from CPP and that results are sustainable. The naturalistic design strengthens the applicability of CPP.

## Introduction

It has been suggested that children under the age of six are disproportionately exposed to potential interpersonal traumatic experiences, such as intimate partner violence (IPV), abuse, and parental loss (United States Department of Health & Human Services, 2022; Woolgar et al., 2021). Due to their dependence on caregivers for safety and emotional regulation, the rapid ongoing brain development, and limited cognitive capacities it has been suggested that young children may be especially vulnerable to such experiences (Dunn et al., 2017; McCrory et al., 2012). Most children demonstrate resilience and return to normal functioning following traumatic events. Nevertheless, it is estimated that up to 20% of trauma-exposed children under the age of six meet the criteria for PTSD (De Young & Landolt, 2018). The prevalence of the diagnoses in this age group appears to be higher following interpersonal and repeated events compared to non-interpersonal or single events (Woolgar et al., 2021). Furthermore, severe non-interpersonal adversities such as accidents and medical trauma can also lead to considerable negative psychiatric and behavioral outcomes in children (Copeland et al., 2007).

Potential traumatic events often affect both children and caregivers, and the impact of trauma during early childhood should be considered in the context of the parent-child relationship (De Young & Landolt, 2018). Studies of the relational context from the perspective of attachment following traumatic events have contributed to the understanding of how childhood trauma involving caregivers may negatively affect the young child (Sroufe et al., 2005). It has been suggested that the quality of attachment is an important factor in children’s capacity to resolve traumatic experiences and the need to recognize that traumatic events can damage existing attachment has been underscored (Chu & Lieberman, 2010). Long-lasting traumatic stress in caregivers can undermine caregiving capability (Fong et al., 2019). Furthermore, caregiver traumatization can cause caregiving limitations that disrupt the development of the young nonexposed child (van Ee et al., 2016).

Caregiving has been described as a complex process reflecting earlier relationship experiences as well as past and current experiences with a child. Hence, caregiving and child attachment are linked and transmitted across generations (Solomon & George, 1996, 2011). Caregiving including for example, frightening and/or frightened, intrusive, withdrawn, or role-confusing behaviors, has been associated with attachment disorganization (Sleed et al., 2021). A disorganized or fearful/avoidant attachment style, related to one or both parents is more frequent in children exposed to potentially traumatic events, such as IPV, abuse, and neglect (Forslund et al., 2021). Further, disorganized attachment seems to increase the risk for post-traumatic symptoms in exposed children (McDonald et al., 2008) and is associated with less positive outcomes in general child development (Fearon et al., 2010).

Research indicates that approximately 50% of young children with trauma symptoms do not recover and will have lasting impairments (Scheeringa et al., 2005). However, most trauma-focused therapeutic methods are designed for school-aged children and youth, and research lacks substantial data concerning children under the age of five years (Gutermann et al., 2016; Sleed et al., 2022). Furthermore, the quality of available research on trauma treatment for young children has several weaknesses, primarily due to small sample sizes and lack of follow-up data (Dorsey et al., 2017; Mavranezouli et al., 2020, Sleed et al., 2022). In Sweden, clinics offering specialized treatment for young children are few and have limited capacity regarding therapeutic competence (Furmark & Neander, 2018). Given the increased risks of exposure, vulnerability, extensive negative consequences, and limited access to trauma-focused treatment, long-term treatment effects for young children are of prime interest, particularly within a naturalistic clinical setting.

### Trauma-Focused Psychotherapy for Young Children

Despite weaknesses in the research on psychotherapeutic interventions for young children, including trauma-focused treatments, its limitations and lack of follow-up data (Dorsey et al., 2017; Mavranezouli et al., 2020), early interventions seem important in preventing negative developmental sequelae, as they promote changes in the relational, physical, and mental health trajectories of trauma-exposed children (Sleed et al., 2022).

Recent reviews provide promising results for some effective treatment methods for traumatized young children, among which Child-Parent Psychotherapy (CPP) is one (Mavranezouli et al., 2020; Sleed et al., 2022). Though several studies show positive results there is a need to further evaluate the applicability of CPP for diverse traumatic events, to explore both individual and treatment predictors, associations between post traumatic symptom improvement in children and caregivers, and long-term outcomes (Alto et al., 2021; Hagan et al., 2017).

### Long-Term Effects of Trauma-Focused Interventions for Young Children

The evidence of the long-term effectiveness of various PTSD treatments for young children is insufficient (Mavranezouli et al., 2020). Follow-ups at 6- and 12 months on children aged 4–13 years having received diverse trauma-focused treatments showed sustained positive outcomes in decreased anxiety (Mannarino et al., 2012; Salloum et al., 2022), improved conduct, reduced externalizing and internalizing problems (McDonald et al., 2006) as well as decreased PTSD-symptoms (Lempertz et al., 2023; Pernebo et al., 2019). Reduced caregiver stress has also been found (Mannarino et al., 2012; McDonald et al., 2006; Salloum et al., 2022). However, contradictory results were reported from a follow-up study, showing that the decrease in child behavioral problems post-intervention was not sustained at the 12-month follow-up (Grip et al., 2012). Thus, the long-term effects of trauma treatments remain unclear.

It is also uncertain which children benefit the most from the treatments at follow-up. One study found that children with higher levels of pre-treatment internalizing, and depressive symptoms seemed to benefit less at a follow-up (Mannarino et al., 2012), while another study found that children with the most severe trauma symptoms benefited the most (Pernebo et al., 2019). Additionally, most follow-up studies concern school-aged children and comprise few children under the age of five. Few studies have evaluated relational or attachment perspectives.

### Long-Term Treatment Effects of Child-Parent Psychotherapy

CPP is one of the few interventions designed for traumatized infants, toddlers, and preschool-aged children, including their caregivers. The dyadic treatment method is trauma-focused and intended to improve parent-child relationships, foster secure attachment, and target contextual factors such as family and cultural values. The intervention addresses traumatic stress reactions in both caregivers and children, promotes affect regulation, changes maladaptive mental representations and behaviors in the child and caregiver, and their relationship. The joint creation of a trauma narrative is central to the emotional processing of adverse experiences and the addressing of challenges within the dyadic interaction. Weekly sessions ranging from 20 or more are suggested; however, treatment length can be briefer depending on the needs of the child and the caregiver. For a thorough description of the treatment method see Lieberman and coworkers (2015). A variety of positive outcomes in children and caregivers after CPP have been shown after adversities such as IPV, abuse, and traumatic loss (Lieberman et al., 2015; Rizo et al., 2011).

The long-term effects of CPP have been evaluated in randomized controlled trials (RCT) (Cicchetti et al., 2011; Ghosh Ippen et al., 2011; Guild et al., 2017, 2021; Lieberman et al., 2006; Stronach et al., 2013; Toth et al., 2015). Analyses of 6-month follow-up data of IPV-exposed children and their mothers indicated that improvements in child behavior problems and maternal psychiatric symptoms were sustained for the CPP group compared to a control group receiving case management and adult individual psychotherapy (Lieberman et al., 2006). A study of Ghosh Ippen and colleagues (2011) revealed that improvements of PTSD- and depression symptoms, co-occurring diagnoses and behavior problems in children, and PTSD-and depression symptoms in the participating mothers, were significantly greater and maintained for a high-risk group (children exposed to four or more traumatic events) compared to a group receiving community services. One year follow ups of maltreated children and their mothers having received CPP showed significant decrease in both child and maternal stress compared to the non-intervention control group (Cicchetti et al., 2011; Toth et al., 2015).

Another study showed that maltreated children receiving CPP were more likely to demonstrate secure attachment at a one-year follow-up, compared to children with caregivers receiving psychoeducational parenting intervention or community standard services (Stronach et al., 2013). A long-term follow-up revealed that children who had received CPP as toddlers with their depressed mothers, showed higher rates of change to secure attachment at age nine, which was associated with higher levels of maternal warmth, compared to a no-treatment control group (Guild et al., 2021). Secure attachment was also associated with lower levels of child anger and problem behavior, not the least reflected in the children’s teachers reports on classroom competence, and positive peer relationships compared to the no-treatment control group (Guild et al., 2017). Thus, the long-term treatment effects from CPP show promise, demonstrating a reduction of child behavior problems, stress, depression, PTSD-symptoms, and maintained secure attachment in children. Furthermore, CPP seem to sustain improvements in stress, PTSD- and psychiatric symptoms among the treated caregivers.

The dissemination of CPP outside the United States of America (USA) is limited. A feasibility study from Sweden (Almqvist et al., 2018; Broberg et al., 2015) showed good acceptance of CPP among therapists and caregivers, and positive tendencies for children’s psychological well-being and general functioning. No signs of a need for cultural adaptations of the method were revealed. A qualitative interview study with caregivers reported positive development regarding child well-being, caregiving capacities, and the caregiver-child relationship after CPP (Norlén et al., 2021). Subsequent exploration of the effectiveness of CPP in a Swedish naturalistic clinical practice setting was proposed (Norlén et al., 2024), along with the long-term treatment effects (the present study).

### Predictors and Outcomes of Treatment

There is limited knowledge about circumstances influencing psychotherapy treatment effects, such as individual conditions for whom treatment works (moderators) and the mechanisms through which treatment achieves effects (mediators). Studies of trauma treatment for children have rarely explored moderators of outcome, likely due to small sample sizes limiting analyses, and reviews report mixed results and conclusions are tentative (Woolgar et al., 2021). A recent review of psychosocial treatment methods for trauma- exposed children by Dorsey and colleagues (2017) found that demographic characteristics, such as the child’s age and gender, do not seem to moderate outcomes, while parental functioning, treatment dose, the use of explicit exposure in treatment, and sudden treatment gains, moderate the effects of traumatic stress symptoms.

A study of moderators of symptom changes during CPP showed that improvements in PTSD-symptoms in parents were associated with reduced symptoms of avoidance and arousal in their child (Hagan et al., 2017). Both parent and child improvements were greater for dyads with fewer parental lifetime stressors, fewer treatment sessions, and more benefitable for girls than boys. Our previous study, exploring the correlations of treatment outcomes in children and caregivers indicated that the effectiveness of CPP for the child was connected to their pre-treatment level of trauma symptoms and the degree to which the relationship with the caregiver improved during treatment (Norlén et al., 2024).

The knowledge of predictors of symptom change at follow-up is limited. In one study, a high degree of pre-treatment trauma exposure was shown to positively moderate outcome, that is, greater reductions of PTSD- and depression symptoms, in both children and mothers receiving CPP at 6-month follow-up compared to a less exposed group (Ghosh Ippen et al., 2011). It should be noted that the less exposed group also benefited from CPP.

Thus, the relationship between moderators and mediators seems ambiguous and complex, complicating the interpretation of whether a condition moderates or mediates outcome, even in large sample studies and RCTs (Kraemer et al., 2002). Regardless, identifying and understanding these aspects and differences in effects is essential to developing treatment methods. Therefore, it has been proposed that identified baseline measures affecting outcome could be referred to as nonspecific predictors of outcome (Kraemer et al., 2002). In the current study, we will proceed from this understanding when exploring possible predictors of treatment outcome at follow-up.

### Aim and Research Questions

This study explored the long-term effects of CPP and potential predictors of outcome in a sample of young children and their caregivers at a 6-month post-treatment follow-up in Swedish naturalistic clinical settings.

The following research questions were addressed:

- What is the long-term effect of CPP on child post-traumatic stress symptoms?
- What are the long-term effects of CPP on caregiver post-traumatic stress symptoms and caregivers’ perceptions of signs of caregiving disorganization in their relationship with their child?
- What are the possible predictive effects of type of child trauma exposure (i.e., interpersonal versus non-interpersonal trauma), trauma symptom severity, and number of therapy sessions, on the outcome of CPP?

## Method

### Study Design

The design was a one-group, pre-post study with a 6-month follow-up in a naturalistic multi-site clinical setting. Assessments were made before therapy (T1), after ≤ 20 dyadic sessions (T2), and at a follow-up six months after the end of the treatment (T3).

### Participants

The sample included 37 children (12 girls and 25 boys) and their caregivers (*n* = 37), 24 biological mothers, 2 biological fathers, and 11 foster mothers. The children were 2 to 6 years old at the start of therapy (*M* = 4.62, *SD* = 1.05). Most of the children were 4–5 years old (*n* = 26), with a smaller part being 3 years old (*n* = 6), 2 years old (*n* = 4), and one child being six years old. The majority of caregivers (81%) were employed, 19% were on long-term sick leave, 54% had a high school diploma, 38% had a university education, and 8% had an elementary school education.

The children’s exposure to potential traumatic events at T1 were assessed by using *The Linköping Youth Lifetime Event Scale – Parent* (LYLES-P) (Nilsson et al., 2012) and *The Violence against the Child (VMB)* (Almqvist & Broberg, 2004; Broberg et al., 2015). Most of the children, 70% (*n* = 26), had been exposed to interpersonal potentially traumatic events e.g., IPV, physical and psychological abuse; 62% (*n* = 23) had been exposed to non-interpersonal potentially traumatic events, e.g., accidents, hospital care, and loss; and 73% (*n* = 27) were reported to have lived with adverse circumstances, e.g., long-term illness, parental separation, divorce or alcohol abuse, and mental health problems within the family. Most of the children had been exposed to several potentially traumatic events according to the LYLES-P, 73% (*n* = 27), to four events and above, 14% (*n* = 5) to three events, and 11% (*n =* 4) had been exposed to 2 events or fewer. The proportion of children being exposed to different types of interpersonal violence according to the VMB and LYLES-P is presented in Table 1.

The caregiver’s exposure to potential traumatic events at T1 was assessed by *The Linköping Youth Lifetime Event Scale* (LYLES) (Nilsson et al., 2010), and *The Violence within the Family* (VIF) (Broberg et al., 2015). The caregivers’ report on the LYLES revealed a mean exposure to potential interpersonal traumatic events at 4.21 (*SD =* 2.17), while the mean exposure to potential non-interpersonal traumatic events was 6.88 (*SD* = 2.48). The mean for adverse childhood circumstances was 2.79 (*SD* = 1.75). About half of the caregivers reported being exposed to IPV as adults (51% physical abuse and 46% psychological abuse) according to VIF.

**Table 1:** Child exposure to type of interpersonal violence measured with *The violence against the child* (VMB) and *The Linköping youth lifetime event Scale – Parent* (LYLES P) and at T1, (*N* = 37)

Type of violence	Frequency % (*n*)
Physical violence	27 (10)
Psychological violence	24 (9)
Sexual abuse	5 (2)
Witnessing IPV	59 (22)
Witnessing sexual violence	13 (5)

### Procedure

Nine clinics offering treatment for trauma-exposed children, among other interventions, contributed to the study. Eight were regular child and adolescent mental health services and one was a non-governmental organization. The treatments were delivered by CPP-certified therapists and therapists in ongoing CPP-training (*N* = 22).

The therapists informed all eligible caregivers and juridical custodians about the study and collected written consent. Caregivers and custodians were informed that participation was voluntary and at any time could be discontinued without affecting the continued treatment. When deemed possible, children were verbally informed of the study and asked for oral consent. The questionnaires were administered by the therapists and, with regard to the children’s young age, were all completed by the caregivers at the time of therapy start (T1), after 20 dyadic sessions or less if the therapy was briefer (T2), and six months after the therapy was closed (T3). The total number of dyadic therapeutic sessions the children and caregivers received varied from 11 to 71 (*M* = 29.68, *Mdn* = 23.50, *SD* = 17.32). Each therapist collected data from one to three dyads in treatment. Data was collected between 2017 and 2023.

The clinics followed their ordinary routines for referral and assessment of children, resulting in a heterogeneous sample of children included in the study, referred by caregivers or other agencies. Inclusion criteria for the children were: (i) exposure to at least one potentially traumatic event (equivalent of Criteria A for posttraumatic stress disorder according to Diagnostic Classification of Mental Health and Developmental Disorders of Infancy and Early Childhood, DC: 0–5 (Zero to Three, 2016), (ii) showing psychiatric symptoms assessed as related to these experiences, (iii) being recommended specialized psychotherapeutic treatment after the clinical assessment and, (iv) being aged 1–6 years at the start of therapy. The inclusion criteria for caregivers, including biological or foster parents, were that they were the non-offending caregiver, responsible for the child’s daily care, able to participate in the treatment and capable of filling in the questionnaires without support from a professional interpreter. Exclusion criteria were if the child showed no need for trauma-focused treatment or if a juridical custodian did not consent to treatment.

### Measures

The same three measures were used from T1 to T3 to assess outcomes in post-traumatic stress symptoms in the child and the caregiver, respectively, as well as signs of caregiving disorganization. All measures were completed by the caregivers.

### Post-Traumatic Stress Symptoms in Children and Caregivers

*The Trauma Symptom Checklist for Young Children* (TSCYC; Briere et al., 2001; Nilsson & Svedin, 2012) is a broad-spectrum caregiver report instrument to assess trauma symptoms in children aged 3–12 years. The TSCYC consists of 90 items and contains nine clinical scales. The scale for Total post-traumatic stress consists of three subscales, i.e., intrusion, avoidance, and arousal; the other scales are anxiety, depression, anger/aggression, dissociation, and sexual concerns. The suggested clinical cutoff is a T-score of 70 indicating PTSD, with T-scores of 65–69 indicating moderate traumatic stress problems. Swedish norm data is available (Nilsson et al., 2012). In this study, raw scores on the total post-traumatic stress subscale were used as the primary outcome. Cronbach’s alpha for the total scale was .87.

*The Impact of Event Scale-Revised* (IES‐R; Weiss, 2004) is a self-report trauma symptoms inventory assessing subjective distress for adults. IES-R contains 22 items and is based on three subscales: intrusion, avoidance, and hyperarousal. Even though the IES-R is not a diagnostic instrument it is suggested that a mean of ≥ 1.8 on the total score indicates PTSD. Analyses are based on raw mean scores as primary outcomes. Cronbach’s alpha for the total scale was .96.

### Signs of Caregiving Disorganization

*The Caregiving Helplessness Questionnaire* (CHQ; Solomon & George, 2011) is a screening tool for disorganized caregiving of children aged 3–11 years. The measure is based on the concept of disorganized caregiving, characterized as a quality of care associated with failed protection of the child. Disorganized caregiving is related to the caregiver’s self-assessment of helplessness in interactions with their child, when they become overwhelmed by fear or lack of control (Solomon & George, 1996). The questionnaire consists of 25 statements on experienced helplessness, fear, and reversed child-caregiver roles, i.e., child caregiving. The CHQ generates a total score of 25–125. High scores indicate disorganized caregiving. Analyses are based on raw mean scores on the total scale as primary outcomes. Cronbach’s alpha for the total scale was .59.

### Data Analyses

All statistical calculations were performed using SPSS Statistics 28.0.1.1 and 29.0.0. Alpha level was set at 0.05. Treatment effects during the follow-up period were explored by applying Piecewise Linear Mixed Models (Piece one: T1-T2, Piece two: T2-T3). Level 1 residuals were assumed to be independent and identically distributed for all models. Model fit was assessed using the Akaike Information Criterion (AIC), with a decrease in AIC of ≤ 2 indicating superior fit (Burnham & Anderson, 2004). Due to the limited sample size, all analyses were based on total scores for the instruments. Within-group effect sizes were calculated using Model-estimated differences in mean values divided by the pretreatment standard deviation, as recommended by Feingold (2009). Possible predictors of outcome were analyzed by adding them as centered covariates in the Linear Mixed Models and interacting them with the time variables (Piece 1 and Piece 2). Predictors were Grand mean-centered, meaning that the Grand mean of all individual’s scores on the predictor were subtracted from the individual’s score on the predictor at baseline. For significant predictors and moderators, as a measure of the effect, we compared estimated slopes between groups (in the case of dichotomous predictors/moderators). For continuous measures, slopes were compared at different values of the predictor/moderator (the sample mean ± 1 standard deviation). Estimated differences in slope were divided by the pooled standard deviation at baseline to achieve a standardized mean difference (Cohen’s d; Feingold, 2009).

### Attrition and Missing Data

Two dyads dropped out from T1 to T2 and at T3 a total of twelve dyads were lost due to different reasons; eight were unreachable, one lived with a protected identity, and in three cases the therapists had ended the employment which hindered contact for follow-up.

Missing data was handled using Restricted Maximum Likelihood Estimation. The proportion of missing data for study variables ranged from 3% to 38%. Analyses of TSCYC are based on 37 children and analyses of IES and CHQ are based on 36 participants, as one caregiver declined completing these measures. A paired samples *t*-test analysis of dropouts showed that it was likely random, as no differences could be noted between dyads who participated in the follow-up and those who did not with respect to demographic and assessment variables.

## Results

**Fig. 1:**
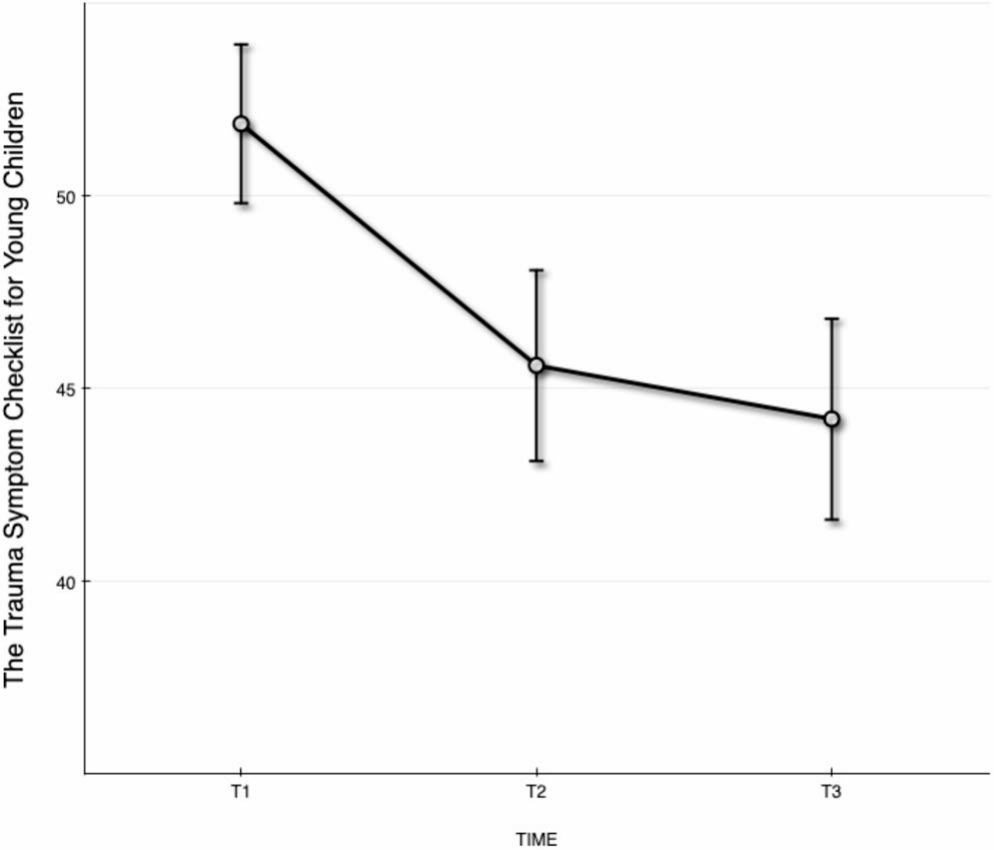
Estimated values with standard error bars on child post-traumatic stress symptoms on the Trauma Symptom Checklist for Young Children (TSCYC) subscale Total posttraumatic stress assessed at T1, T2, and T3

**Fig. 2:**
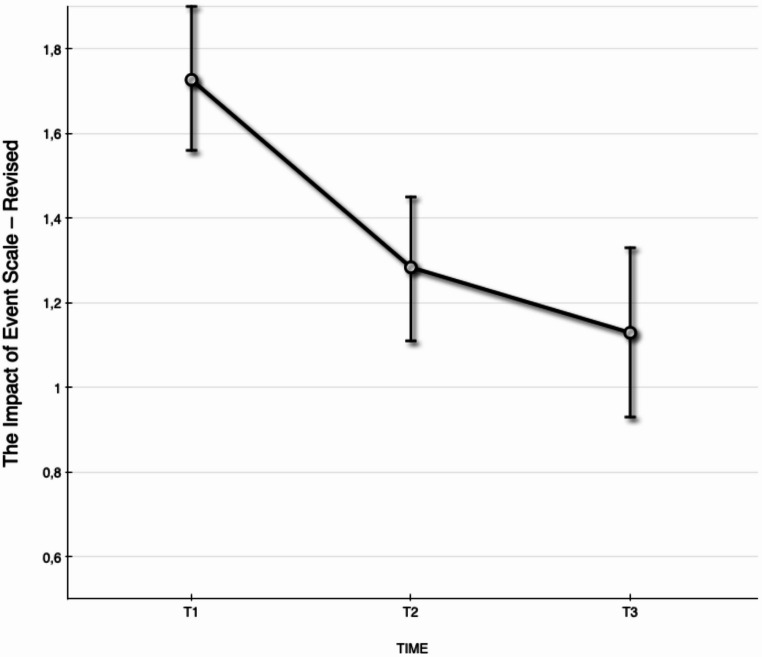
Estimated values with standard error bars on caregiver post-traumatic stress symptoms on the Impact of event Scale - Revised (IES-R) total scale of posttraumatic stress assessed at T1, T2, and T3

**Fig. 3:**
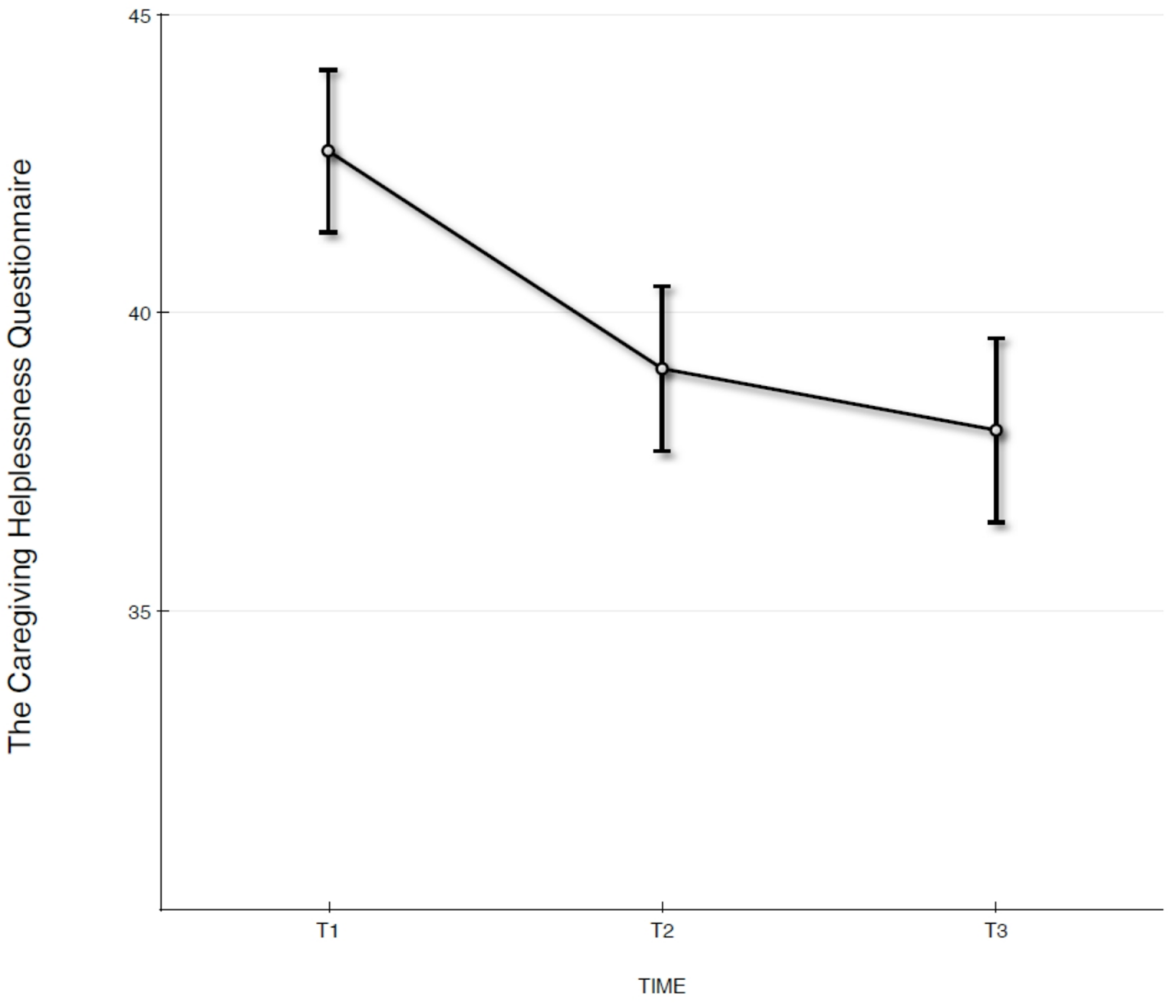
Estimated values with standard error bars on signs of caregiving disorganization on the Caregiver Helplessness Questionnaire (CHQ) total scale assessed at T1, T2, and T3

Analyses presented in this section include data from T1, T2, and T3. Observed values and the number of observations for the TSCYC, IES, and CHQ at T1, T2, and T3 are presented in Table 2. Estimated values are presented in Figs. 1, 2 and 3.

**Table 2:** Observed values and number of observations for outcomes in post-traumatic stress symptoms in children with the trauma symptom checklist for young children (TSCYC), post-traumatic stress symptoms in caregivers with the impact of event Scale-Revised (IES-R), and signs of caregiving disorganization with the caregiver helplessness questionnaire (CHQ) at T1, T2 and T3

	T1	T2	T3
Measure	*M* (*SD*)	*M* (*SD*)	*M* (*SD*)
TSCYC PTS Tot	51.97 (12.35)	45.49 (15.58)	42.00 (12.78)
*n*	36	35	25
IES-R Tot	1.72 (1.05)	1.23 (1.07)	1.03 (0.83)
*n*	36	33	23
CHQ Tot	42.72 (7.60)	39.12 (9.34)	37.89 (7.27)
*n*	36	34	25

### Changes in Children’s Post-Traumatic Stress Symptoms from Pre-Intervention to the 6-Month Follow-Up

Changes in children’s post-traumatic stress symptoms assessed by the TSCYC total post-traumatic stress scale as the primary outcome measure, showed sustained positive effects from T1 to T3. The best-fitting model was a linear model with random intercept and random Piece 1 slopes. Random effects were assumed independent with different variances. Children’s post-traumatic stress symptoms showed no significant changes during the follow-up period (T2 – T3), estimate = −1.38 [95% CI: −4.90, 2.13], F_1,32_ = 0.645 *p* =.43, indicating an effect size of *d* = −0.11. From T1-T3, a medium effect size was found (*d* = 0.62). See Fig. 1 for a graphical representation of predicted values. Conversion of observed raw scores to T-scores showed that 78% of the children had a T-score ≥ 70 at T1, indicating possible PTSD. This percentage dropped to 51% at T2 and 32% at T3.

### Changes in Caregiver’s Post-Traumatic Stress Symptoms from Pre-Intervention to the 6-Month Follow-Up

Changes in caregivers’ post-traumatic stress symptoms assessed by the IES-R revealed sustained positive effects from T1 to T3. For IES-R, the best fitting model was with random intercept and no random slopes. Changes in caregivers’ post-traumatic stress symptoms revealed sustained positive effects during the follow-up period (T2 – T3), estimate: −0.16 [95% CI: −0.53, 0.22], F_1,57_ = 0.67, *p* =.42, indicating an effect size of *d =* 0.15, resulting in a medium effect size from T1 to T3 (*d* = 0.57). See Fig. 2 for a graphical representation of predicted values.

A mean of ≥ 1.8 on the IES-R total score is suggested to indicate post-traumatic stress disorder. At T1 nearly half of the caregivers (47%) had a score over ≥ 1.8, while the proportion decreased to 30% at T2, and 17% at T3.

### Changes in Signs of Caregiving Disorganization from Pre-Intervention to the 6-Month Follow-Up

Changes in caregiver-perceived signs of caregiving disorganization from T1 to T3 assessed by the CHQ showed persistent positive effects at T3. For CHQ, the best fitting model was with random intercept and no random slopes. No significant changes in perceived signs of caregiving disorganization were observed during the follow-up period (T2–T3), estimate = −1.03 [95% CI: −4.09, 2.04], F_1,61_ = 0.447, *p* =.51). The change during the follow up period indicates an effect size of *d = -*0.14, showing a medium effect size for change from T1 to T3 (*d =* −0.62).

### Baseline Predictors of Outcomes

**Fig. 4:**
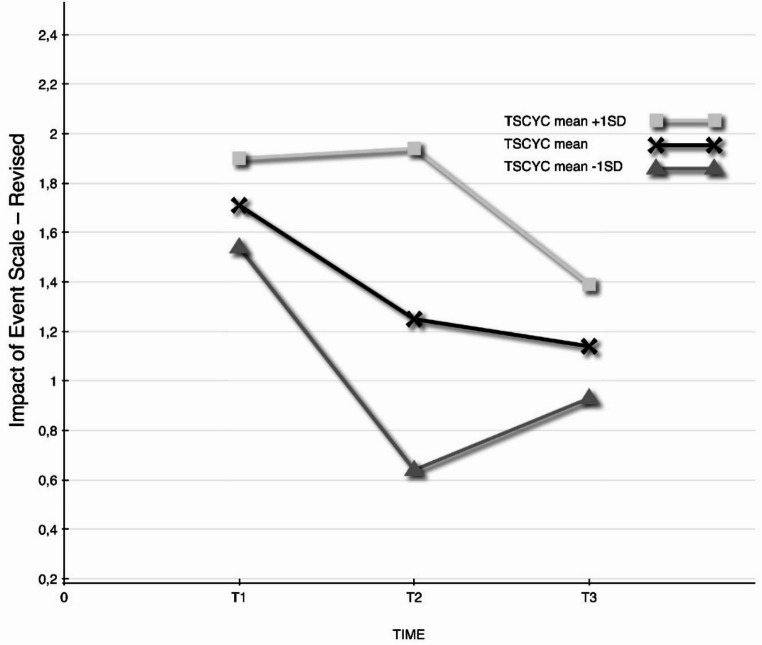
Interaction of child and caregiver PTSD symptoms outcomes (TSCYC and IES-R) assessed at T1, T2, and T3

Exploratory analyses of possible predictors of outcome investigated type of trauma exposure (i.e., interpersonal versus non-interpersonal trauma), baseline trauma symptom severity in both child and caregiver (measured by TSCYC and IES-R), and number of therapy sessions, on all three outcome measures. Baseline variables were not used as predictors since they were part of the growth model in the linear mixed model, and this would violate model assumptions. No significant predictors were found concerning the type of trauma (interpersonal versus non-interpersonal) or number of dyadic sessions. However, one predictor showed a significant interaction between baseline TSCYC and Piece 1 on the IES-R, meaning that higher trauma symptoms in the child at T1 predicted less change in caregiver trauma symptoms during the treatment phase, estimate = 0.03, [95% CI: 0.0, 0.05], F_1,51_ = 4.16, *p* =.047. This means that for each point the child was over the mean value of TSCYC at baseline, caregiver IES-R scores changed 0.03 points less during the treatment phase. Figure 4 illustrates the different growth rates in trauma symptoms in both phases, for caregivers whose children present relatively high (+ 1 SD), average and relatively low scores (− 1 SD) on TSCYC. The standardized mean difference in change during the treatment period between the group − 1 SD below the mean and the group + 1 SD above the mean on baseline TSCYC was *d* = 0.89. It should be noted that parents of children with high TSCYC scores had more positive trajectories during the follow-up period (T2-T3) (*d* = 0.75), resulting in similar end-scores. However, this difference was not significant. Given the small sample size this might well be due to lack of statistical power.

## Discussion

In a Swedish sample, the present study aimed to explore the long-term effectiveness of CPP at a 6-month follow-up in a naturalistic multi-site setting. Outcomes were examined in a sample of young, traumatized children aged 2–6 years, receiving dyadic treatment together with their caregivers, including both biological and foster parents. Since the method was recently introduced in Sweden, it was deemed essential to carefully examine its functioning in this new context, with the naturalistic approach offering valuable insights in this regard. Notably, this was the first time an effectiveness study was conducted outside the United States of America. Consistent with a previous follow-up of CPP (Ghosh Ippen et al., 2011), the results indicated maintained treatment gains for both child and caregiver post-traumatic stress symptoms at follow-up. In line with a previous study evaluating long-term effects on attachment at 12-month follow-up (Stronach et al., 2013), the present study showed a decrease in self-reported signs of caregiving disorganization at the time of the follow-up. Conducted in a new sociocultural context, our study—though limited—adds supporting evidence to previous studies showing that CPP can be an effective treatment for young children and caregivers exposed to various types of adversity and traumatic events.

Extensive comparisons with previous research are limited due to various study designs and measurements. However, the results of the current study seem to mirror the positive long-term impact on child-caregiver attachment previously demonstrated (Guild et al., 2017, 2021; Stronach et al., 2013). The importance of secure attachment for future healthy child development is a well-established finding. The beneficial long-term effect of supporting the young child’s relationship with the caregiver has been emphasized in PTSD treatment guidelines (Landolt et al., 2017) and findings in meta-analytic research (Gutermann et al., 2016). In CPP, enhancing dyadic emotional regulation and the creation of a joint trauma narrative with children and caregivers are examples of how attachment relationship improvement seems to be achieved. By supporting caregivers and children in sharing traumatic experiences, painful emotions, and developing mutual regulation within the dyad, CPP can enhance the sense of safety and protection for both parties, improve communication, and repair conflict, stress and dysregulation often associated with trauma.

The present study confirms the long-term stability of the positive treatment outcomes indicated in a previous effectiveness study in the Swedish clinical context (Norlén et al., 2024). Sustainable treatment effects of CPP when provided in a naturalistic clinical context, as indicated in our study, illuminates the possibility of providing effective attachment- and trauma-informed interventions for young children with trauma-related symptoms and their caregivers. Early onset interventions are critical, as young children appear to be particularly vulnerable to traumatic experiences and may be at risk of significant negative developmental outcomes.

Most of the children in the present sample had a high degree of exposure to potentially traumatic events and exhibited symptoms indicating PTSD at T1. Given that as many as 86% (*n* = 32) of the children had been exposed to three or more potentially traumatic events, it was expected that the type of event (interpersonal or non-interpersonal) or the severity of trauma related symptoms would have a predictive impact on the outcome. However, our analyses did not reveal any such associations. These relationships should be further examined in future studies, also as existing research on predictors has yielded inconclusive results. Even though most children showed a significant decrease in symptoms during treatment, a relatively high proportion still showed many symptoms at follow-up. This reveals the need for long-term evaluation after treatment, as well as more studies to investigate the treatment effects more closely.

The exploratory analyses examining predictors in the current study, including type of child trauma exposure, degree of child post-traumatic stress symptoms, and the number of therapy sessions, did not affect the outcome. This may be a reflection of the small sample size, limiting possible analyses as also found in previous research on outcome predictors in child trauma treatment (Woolgar et al., 2021). A further study, involving a larger sample might enhance the ability to investigate predictors.

However, one significant predictor was revealed, where a higher degree of pre-treatment trauma symptoms in the child seems to predict less reduction in caregiver traumatic stress symptoms. This result should be interpreted with caution, since several exploratory analyses were conducted on a small sample and no correction for multiple significance tests were performed and needs to be tested in a larger sample. Nevertheless, the finding could be interpreted with the clinical implication that the caregiver’s traumatic burden and recovery are affected by that of the child. It has previously been suggested that improvement in the child’s symptoms constitutes a positive factor in maternal symptom decline in a 6-month follow-up of CPP (Lieberman et al., 2006).

### Limitations

The primary limitation of the current study is the small sample size which limits the statistical analyses as well as the interference. Nevertheless, the significant results obtained were consistent across measures and support the conclusion that the positive effects of CPP are sustainable over time for both children, caregivers and their relationship. Additional limitations were the lack of a control group and the reliance on caregiver reports, which increases the risk of response bias, though these are common constraints in naturalistic studies. The naturalistic design necessitated a simple and achievable measurement approach to examine the complex link between caregiving representation and child attachment. For example, the non-direct assessment of caregiving disorganization entails risks for response bias. The importance of screening tools for researchers and practitioners to assess risks in caregiving representations has previously been emphasized (Sleed et al., 2021). CHQ is a screening tool intended to assist researchers in studying parent-child relationships from an attachment perspective (Solomon & George, 2011). Hence, signs of caregiving disorganization are theoretical interpretations of disorganized child attachment as the two systems are reciprocal (Solomon & George, 1996). CHQ is less costly and time-consuming compared to standardized observational methods. However, in the present study CHQ demonstrated weak reliability, which might be attributable to the Cronbach’s Alpha being calculated using the total score, despite the measure being multi-dimensional rather than unidimensional. This finding may also reflect issues with the applicability of the instrument in the current sociocultural context and underscores the need of a future Swedish validation study.

Assessing traumatic stress symptoms in young children is multifaceted and should ideally cover diverse types of reactions including relational aspects (Moner et al., 2022). The measurement used in this study (TSCYC) is designed to evaluate several domains of trauma symptoms but is restricted to tentative PTSD diagnosis. TSCYC is not validated for children under the age of three. However, at T1, four children in the current study were younger than three. While one child was not assessed at that timepoint, assessments were deemed clinically relevant for the remaining three. Furthermore, clinical cut off scores for children under the age of five are unclear and low scores can imply significant problems for a young child (Nilsson et al., 2012). It has been shown that impairment rates, e.g. restricted participation with family and peers, can be higher than PTSD diagnostic rates and indicate the need for treatment in preschool children (Scheeringa et al., 2005). Thus, the use of more extensive and independent assessment methods of psychological symptoms and attachment quality would contribute to a more thorough examination and understanding of the long-term effects of CPP.

A longer follow-up period might have contributed to the comprehensive understanding of possible long-term gains. However, the relatively large attrition rate in this study reveals challenges in longitudinal clinical studies. Similar attrition rates in previous studies of CPP ranging from 14 to 40% have been interpreted as a function of intervention studies in general rather than of the CPP method in particular (Alto et al., 2021). Despite the abovementioned limitations, the naturalistic setting of our study strengthens the external validity and the generalizability to similar clinical contexts and might serve as a starting point for further and more extensive research. The results also indicate that further dissemination of CPP in a Swedish clinical context can be supported.

### Clinical Implications

The results of the study indicate that both traumatized children and caregivers and their attachment relationship benefit from CPP and that benefits persist beyond the conclusion of therapy. Both positive effects in terms of reduced child and caregiver post-traumatic stress symptoms and signs of disorganized caregiving were maintained when followed up six months after the intervention. The observed predictive inhibitory effect of child trauma symptom severity on caregiver traumatic stress recovery underscores the clinical importance of continuously addressing caregiver mental health when treating young children, as persistent traumatic stress can limit caregiving capacity. The finding also supports the relevance of a core domain of CPP: helping caregivers develop awareness of how their own histories may influence parenting behaviors and affect their ability to understand and meet their child’s needs.

The naturalistic design mirrors the Swedish clinical context and strengthens the applicability of CPP for traumatized young children in Swedish clinics providing psychotherapeutic treatment. Since effective treatment options are often lacking for young children, the findings are encouraging regarding further dissemination of the method. The stable positive effects indicated in the study support the value of offering early interventions and may provide valuable insights for clinics facing limited resources.

## Data Availability

Not Applicable. No supporting data available.
